# Moderate intensity walking exercises reduce the body mass index and vascular inflammatory factors in postmenopausal women with obesity: a randomized controlled trial

**DOI:** 10.1038/s41598-023-47403-2

**Published:** 2023-11-17

**Authors:** Woo-Hyeon Son, Hyun-Tae Park, Byeong Hwan Jeon, Min-Seong Ha

**Affiliations:** 1 Institute of Convergence Bio-Health, Dong-A University, 26 Daesingongwon-ro, Seo-gu, Busan, 49201 Republic of Korea; 2 Graduate School of Health Care and Sciences, College of Health Science, Dong-A University, 37, Nakdong-daero 550beon-gil, Saha-gu, Busan, 49315 Republic of Korea; 3 Department of Sports and Health Science, College of Arts, Kyungsung University, 309, Suyeong-ro, Nam-gu, Busan, 48434 Republic of Korea; 4 Laboratory of Sports Conditioning: Nutrition Biochemistry and Neuroscience, Department of Sports Science, College of Arts and Sports, University of Seoul, 163 Seoulsiripdaero, Dongdaemun-gu, Seoul, 02504 Republic of Korea

**Keywords:** Biochemistry, Immunology, Physiology, Cardiology, Endocrinology, Health care

## Abstract

Postmenopause, the secretion of female hormones changes, causing excessive fat accumulation in the body and leading to chronic inflammation, which increases the incidence of cardiovascular diseases (CVD). Walking is an easily accessible daily exercise and effective non-pharmacological treatment for reducing obesity and the incidence of CVD. The aim of this study was to investigate the effect of moderate intensity walking exercises on body composition, vascular inflammatory factors, and vascular endothelial growth factor (VEGF) in postmenopausal women with obesity. Twenty-six older postmenopausal women with obesity (ages 68–72) were randomly assigned to control (*n* = 12, BMI 26.06 ± 1.37) or exercise (*n* = 14, BMI 26.04 ± 1.94) groups. Following a 12-week moderate intensity walking exercise program, we measured the participants’ body composition with an InBody S10 analyzer and assessed blood sera using enzyme-linked immunosorbent assays. There was a significant clustering by weight (*p* < 0.01), body mass index (*p* < 0.01), percentage body fat (*p* < 0.001), high-sensitivity C-reactive protein (*p* < 0.05), interleukin-6, and tumor necrosis factor-α (*p* < 0.05) being significantly decreased in the exercise group. Although VEGF levels did not change significantly, a tendency to increase was observed in participants that exercised. Our results indicate that walking exercise may help prevent CVD in postmenopausal women with obesity by reducing obesity and vascular inflammatory factors.

## Introduction

Menopause is a naturally occurring aging process in women, and cardiovascular diseases (CVD) notably increase after menopause^1^. In addition, postmenopausal hormonal changes, such as a deficiency in the female hormone estrogen, accelerate fat accumulation in the abdomen, possibly leading to obesity^2^. A previous comparative study reported that the body fat mass is higher after than before menopause^3^. Longitudinal Study reported that changes in body weight were associated with an increased risk of cardiovascular diseases^4^. Cross-sectional studies reported associations between obesity and inflammatory factors^5^. The Korea National Health and Nutrition Examination Survey between 2016 and 2017 reported that obesity is a risk factor for the elevation of inflammatory markers in postmenopausal women^6^. When excess fat accumulates in the body, the inflammatory response intensifies, leading to chronic inflammation. Chronic inflammatory conditions in women after menopause in turn, increase the incidence of CVD^7,8^.

High-sensitivity C-reactive protein (hs-CRP), interleukin-6 (IL-6), and tumor necrosis factor-α (TNF-α) are crucial vascular inflammatory factors^9^. They have at high levels of hs-CRP^10^, IL-6^11^, and TNF-α^12^ in obese individuals. Inflammation factors known to play a key role in the pathogenesis of arteriosclerosis and CVD^13^. Hs-CRP is produced in the liver upon stimulation by TNF-α and IL-6^9^, and its levels are used to measure various inflammatory responses and diseases^14^. In particular, hs-CRP serves as an index indicating the degree of the inflammatory response of atherosclerotic vascular^15^. Hs-CRP inhibits the stimulation of angiogenesis by vascular endothelial growth factor (VEGF), with the latter playing a vital role in forming new capillaries by promoting endothelial cell proliferation^16,17^. In addition, blood VEGF levels were reported to be low in obese people in studies on the relationship between body mass index and VEGF^18^. Obesity worsens the condition of vascular, such as reducing the density of blood vessels and capillary activity. As such, an increase in inflammatory factors caused by obesity increases the risk of CVD in women who have undergone menopause.

Regular exercise is recommended as a non-pharmacological method to reduce this risk of cardiovascular disease. A review and meta-analysis study by Zheng et al*.*^19^ reported that regular aerobic exercise effectively reduces inflammatory cytokine levels, thereby improving resistance to cardiovascular disease^20^. Among various aerobic exercises, walking is a physical activity with low impact compared to running. With less impact on the musculoskeletal system and joints, it is highly recommended for women who are obese, older or possess weak physical strength^21^. Walking is also an accessible form of exercise that increases physical activity in daily life, and the American Heart Association recommends walking over 7000 steps daily. In previous cohort studies reported that people who walked a minimum of 7000 steps per day had a 50–70% lower risk of mortality compared to those who walked fewer than 7000 steps per day^22^. In addition, as the number of steps increases by 1000, the levels of IL-6 and CRP decrease by 13%^23^. In a cohort study demonstrated with every increase of 1000 steps, there was a reduction in the levels of inflammation within the body in elderly individuals^24^.

The objective of the present study has been refined as follows: This research endeavored to assiduously explore the consequences of engaging in walking exercise of moderate intensity quantitatively defined as a regimen involving 7000–9999 steps per day at a pace of 100 steps per minute^22^ on several health parameters, including body mass index, hs-CRP, IL-6, TNF-α, and VEGF levels, among postmenopausal women navigating through obesity. A comparative analysis was implemented against a cohort of demographically analogous individuals who refrained from regular exercise. The guiding hypothesis of this research speculated that a walking exercise protocol, characterized by its moderate intensity, would elicit tangible enhancements in vascular inflammation and VEGF levels, as evidenced by a juxtaposition with the outcomes derived from the non-exercising cohort.

## Methods

### Study design

A randomized controlled trial was conducted to confirm the effect of moderate intensity walking for 12 weeks. The block randomization method in SPSS Statistics for Windows, Version 22.0 (IBM Corp., Armonk, NY, USA) was used. The 26 participants were divided into either a control (*n* = 12, age 69.91 ± 1.14, BMI 26.06 ± 1.37) or an exercise (*n* = 14, age 70.17 ± 1.21, BMI 26.04 ± 1.94) group. Anthropometrics and Blood collection were performed on each participant at the same time in the morning (08:00 AM, ± 1 h) pre and post the 12-week intervention. Individuals in the control group had not exercised regularly (Table 1). Participants were restricted from taking any medications and engaging in exercises that could influence the trial results during the study period. Subsequent to these restrictions, safety matters were additionally verified through medical examinations.

**Table 1:** The number of steps taken by the participants.

Variables	Exercise (n = 14)	Control (n = 12)
Walking step	9106.90 ± 1191.44***	1960.91 ± 90.60
Sedentary time (min)	831.60 ± 96.33**	1202.91 ± 125.17

*EX* exercise group, *CON* control group.

Values are presented as mean ± standard deviation.

**p < 0.01, ***p < 0.001 EX vs. CON.

### Participants

**Figure 1:**
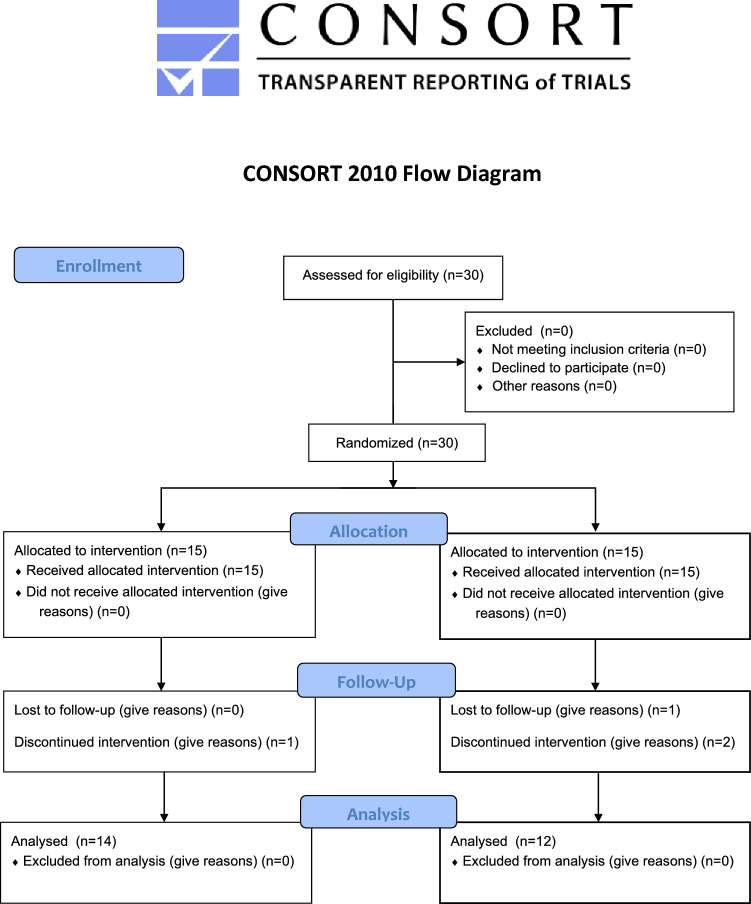
CONSORT flow diagram for the individual randomized controlled trial.

Participants were recruited with flyers in a metropolitan city in South Korea at a senior community center and a singing class from November 1, 2021, to December 1, 2022. The inclusion and exclusion criteria were set to ensure that the participants had not received drug treatments in the past six months, did not exercise regularly, and did not possess known musculoskeletal disorders. We used G-power version 3.1 software (Kiel University, Kiel, Germany) with an effect size of 0.25 (default), a significance level of 0.05, and a power of 0.70 as parameters to determine the effective sample size required, which was calculated as 26 participants. Therefore, a total of 30 people were recruited for this study, considering potential dropouts. Our participants were postmenopausal women with obesity (68–72 years old) who were fully aware of the purpose and contents of the study and submitted informed consent for participation. Among them, three people from the control group and one from the exercise group discontinued due to personal circumstances, rendering a final sample population of 26 individuals (Fig. 1). Table 2 summarizes the characteristics of all participants. Written signed consent was collected from each participant before the start of the study. This study received approval from the Institutional Review Board designated by the Ministry of Health and Welfare of Korea (Approval Number: 2-1040709-AB-N-01-202109-HR-066-0 4) and was conducted in strict adherence to the principles of the Helsinki Declaration and ethical guidelines for research. Furthermore, this trial was retrospectively registered with the Clinical Research Information Service (CRIS), Republic of Korea (Registration Number: KCT0008621, Date of Registration: 17/07/2023, Website: https://cris.nih.go.kr).

**Table 2:** Characteristics of the participants at baseline.

Variables	EX (n = 14)		CON (n = 12)	
	Pre	Post	Pre	Post
Age (years)	70.2 ± 1.21		69.9 ± 1.14	
Height (cm)	154.69 ± 4.15		155.82 ± 2.52	
Weight (kg)	62.23 ± 4.19	61.26 ± 3.80	63.22 ± 2.49	63.44 ± 2.17
BMI (kg/m^2^)	26.04 ± 1.94	25.63 ± 1.71	26.06 ± 1.37	26.15 ± 1.26
Body fat (%)	37.33 ± 3.37	36.12 ± 3.21	37.0 ± 1.57	37.11 ± 1.77
Skeletal muscle mass (kg)	20.18 ± 1.50	20.41 ± 1.58	20.98 ± 1.57	20.85 ± 1.57

Values are presented as mean ± standard deviation.

*EX* exercise group, *CON* control group, *BMI* body mass index.

### Exercise program

The walking route was pre-determined and close to the participants' residential areas. Each exercise session started with a 20-min warm-up. Participants performed a walking exercise at an intensity between 64–76% of their maximum heart rate (HRmax) measured using a Polar RS400sd monitor (APAC model 90026360; Polar Electro, Bethpage, NY, USA). The baseline cadence was 100 steps/min^25,26^, adjusted according to the walking conditions in question. For their safety, the wearable heart rate monitor sounds an alarm when your heart rate exceeds 64–76% of your HRmax. They activated heart rate monitor alarms to control heart rate at 64–76% HRmax (i.e., 110–130 b/min) during walking sessions^27^. Haptic vibratory feedback was sent to participants to help maintain walking intensity. The control group maintained their conventional physical activity and diet. We monitored compliance once a week via a Fitbit Charge 4 activity tracker (Fitbit, San Francisco, CA, USA), which participants had to wear throughout the day except for sleeping and bathing.

### Anthropometrics

The participants wore simple clothes that contained no metal to ensure accurate measurements and minimum of 8 h of fasting to retain an empty stomach until measurements were taken. Height was measured using a portable extensometer InlabS50 (Biospace Corp., Seoul, Korea). Body composition was determined using Inbody S10 (Biospace Corp., Seoul, Korea) before and after 12 weeks, evaluating weight (kg), percentage of body fat (PBF; %), and skeletal muscle mass (SMM; kg). Body mass index (BMI) was calculated by taking the body mass divided by height squared (kg/m^2^).

### Blood sampling and analysis

All participants were instructed to fast for ≥ 8 h before sample collection. In the morning, 8 − 10 a.m., 10 mL of blood was collected from the antebrachial vein by a clinical pathologist. The blood was centrifuged at 3000 rpm for 10 min in Combi-514R (Hanil, Seoul, Korea) for further analysis. An enzyme-linked immunosorbent assay (ELISA) was used to measure circulating plasma levels of TNF-α and IL-6 (Quantikine HS Human TNF-α and Quantikine HS Human IL-6 kits, respectively; R&D Systems, Minneapolis, USA). For data acquisition and analysis of the ELISA assays, we used a VersaMax absorbance microplate reader (Molecular Devices, Sunnyvale, CA, USA). Blood CRP was analyzed by turbidimetric immunoassay using a CRP4 kit (Roche, Basel, Switzerland) Cobas 8000 equipment (Roche). Serum VEGF was analyzed using a Human VEGF Quantikine ELISA kit (R&D Systems) and the VersaMax absorbance microplate (Molecular Devices).

### Data analysis

The data were processed and analyzed using SPSS 27.0 (IBM, New York, NY, USA). The means and standard deviations of data were calculated. First, by conducting a normality test on our research results, we confirmed that they were normally distributed. To investigate the effect of 12 weeks of moderate intensity walking exercises on changes in vascular inflammatory factors and VEGF, we set the treatment groups (control or exercise) and time (pre- and post-treatment) as independent variables and performed two-way repeated measures ANOVA. Bonferroni post hoc analysis was used. We set statistical significance at *p* < 0.05.

### Ethics approval and consent to participate

The study protocol was approved by the Ministry of Health and Welfare of Korea (2-1040709-AB-N-01-202109-HR-066-04) and was performed in compliance with the Helsinki Declaration and ethical research principles. Written signed consent was collected from each participant before the start of the study. This trial was retrospectively registered in the Clinical Research Information Service (CRIS) (Republic of Korea, KCT0008621, 17/07/2023, https://cris.nih.go.kr).

## Results

### Effect of moderate intensity walking exercises on changes in body composition

**Figure 2:**
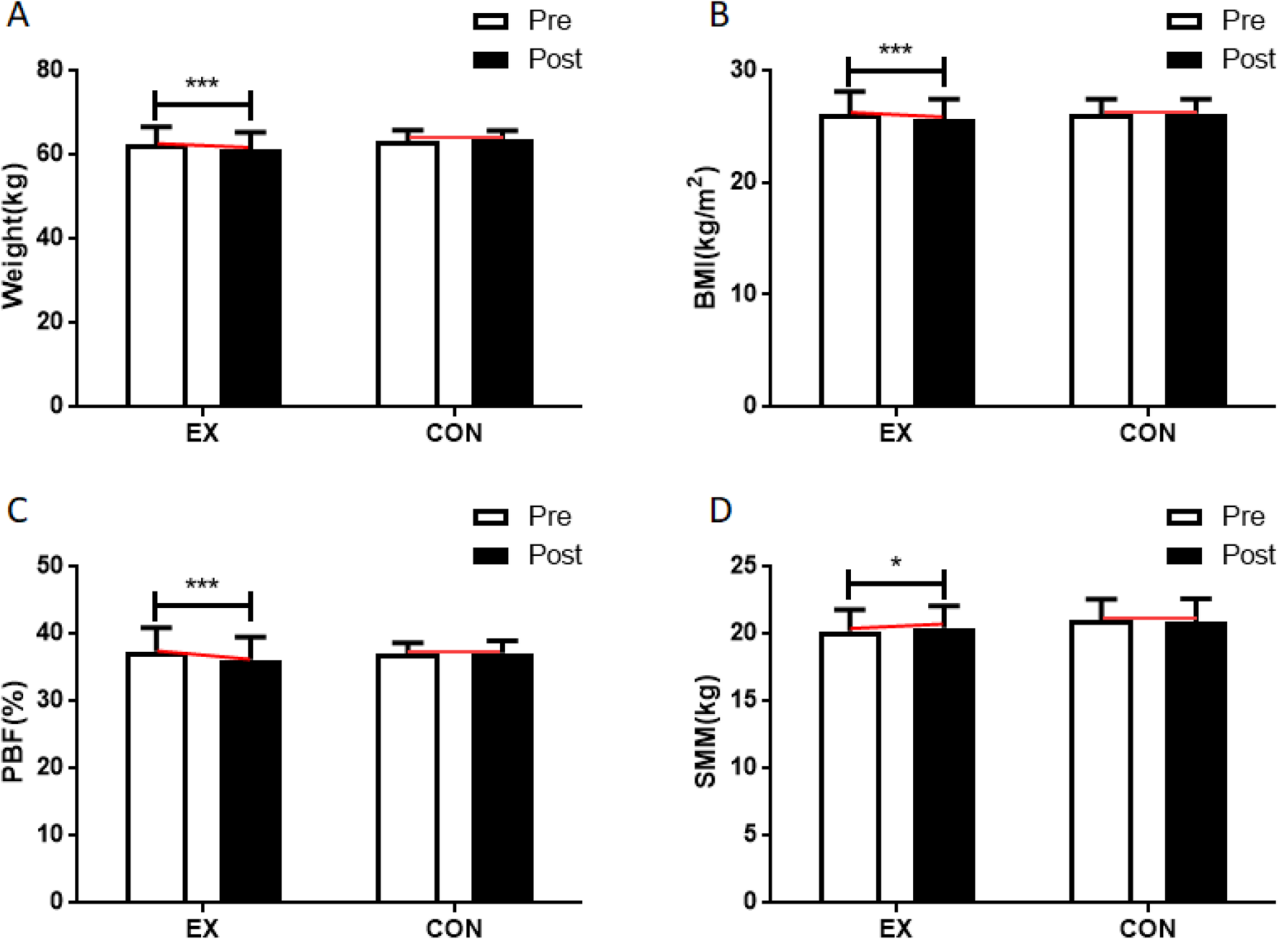
Effect of 12 weeks of moderate intensity walking exercise on body composition in postmenopausal women with obesity. After the intervention, compared with the baseline values, (**A**) Weight levels were decreased in the moderate intensity walking exercise group and (**B**) BMI levels were decreased in the moderate intensity walking exercise group, (**C**) PBF levels were decreased in the moderate intensity walking exercise group, (**D**) SMM levels were increased in the moderate intensity walking exercise group. Data are presented as the mean ± standard deviation; *p < 0.05, ***p < 0.001 before vs. after intervention. *BMI* body mass index, P*B*F percentage of body fat, *SMM* skeletal muscle mass.

Walking exercises affected changes in body weight (interaction effect: *F* = 7.446, *p* = 0.013; time effect: *F* = 2.971, *p* = 0.099; group effect*: F* = 1.259, *p* = 0.274). The post hoc analysis of body weight by pre- and post-treatment period revealed a significant decrease in body weight in the walking group (*F* = 10.363, *p* = 0.004). Body mass index (BMI) was also affected by walking exercises (interaction effect: *F* = 9.203, *p* = 0.006; time effect*: F* = 3.894, *p* = 0.062; group effect: *F* = 0.152, *p* = 0.701), with a significant decrease in BMI in participants of the exercise group (*F* = 13.105, *p* = 0.002). Body fat percentage was equally changed through the exercises (interaction effect: *F* = 11.181, *p* = 0.003; time effect: *F* = 7.804, *p* = 0.011; group effect:* F* = 0.085, *p* = 0.773), with participants who did walking exercises showing a significant decrease in body fat percentage at the conclusion of the study (*F* = 19.689, *p* = 0.000). Lastly, skeletal muscle mass was affected by walking exercises (interaction effect: *F* = 5.656*, p* = 0.027; time effect: *F* = 0.435, *p* = 0.517; group effect: *F* = 0.852, *p* = 0.367), as evident in its significant increase in the walking group (*F* = 4.824, *p* = 0.039). The above results are summarized in Fig. 2 and Table S1.

### Effect of moderate intensity walking exercises on vascular inflammation factors

TNF-α was affected by walking exercises (interaction effect: *F* = 6.895, *p* = 0.016; time effect: *F* = 1.111, *p* = 0.304; group effect: *F* = 1.887, *p* = 0.184). Post hoc analysis of TNF-α levels before and after the experiment showed a significant decrease of this inflammation factor in the walking group (*F* = 7.078, *p* = 0.015). However, IL-6 was not affected by the exercises (interaction effect: *F* = 5.792, *p* = 0.025; time effect: *F* = 0.007, *p* = 0.933; group effect: *F* = 0.286, *p* = 0.598), at the conclusion of the experiment, a significant difference was observed in the interaction effect, but no significant differences were detected at the main effect levels.

**Figure 3:**
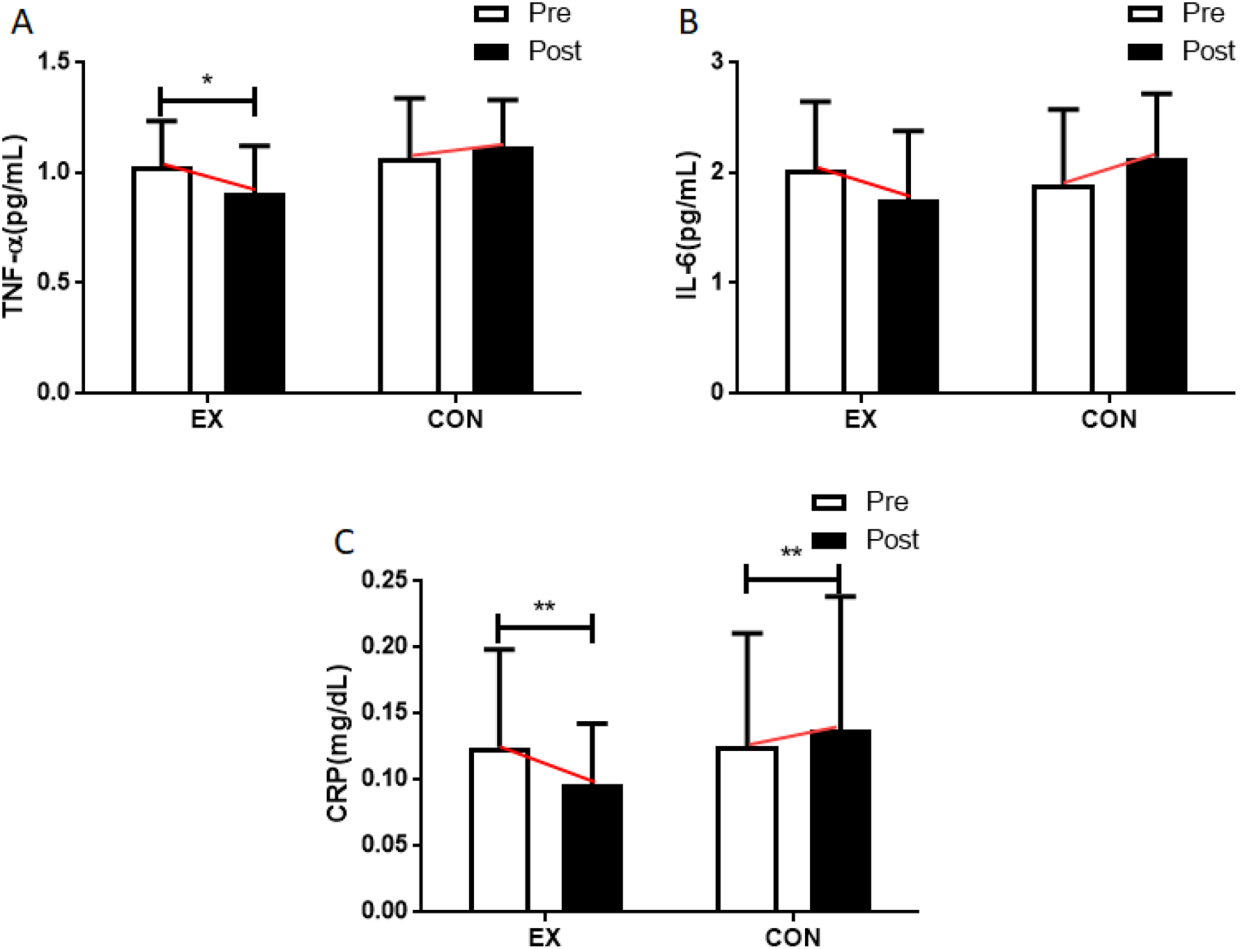
Effect of 12 weeks of moderate intensity walking exercise on vascular inflammation factors in postmenopausal women with obesity. After the intervention, compared with the baseline values, (**A**) TNF-α levels were decreased in the moderate intensity walking exercise group and (**B**) IL-6 levels were not changed in all groups, (**C**) CRP levels were decreased in the moderate intensity walking exercise group and increased in the control group. Data are presented as the mean ± standard deviation; *p < 0.05, ** *p* < 0.01 before vs after intervention. *TNF-α* tumor necrosis factor-alpha, *IL-6* interleukin-6, *CRP* C-reactive protein.

The walking program did affect hs-CRP levels in the blood (interaction effect: *F* = 7.493, *p* = 0.012; time effect: *F* = 1.268, *p* = 0.273; group effect: *F* = 0.450, *p* = 0.510), which were significantly decreased post-treatment in participants in the walking group (*F* = 7.802, *p* = 0.011). Figure 3 and Table S1 illustrates the above findings.

### Effect of moderate intensity walking exercise on vascular endothelial growth factor

**Figure 4:**
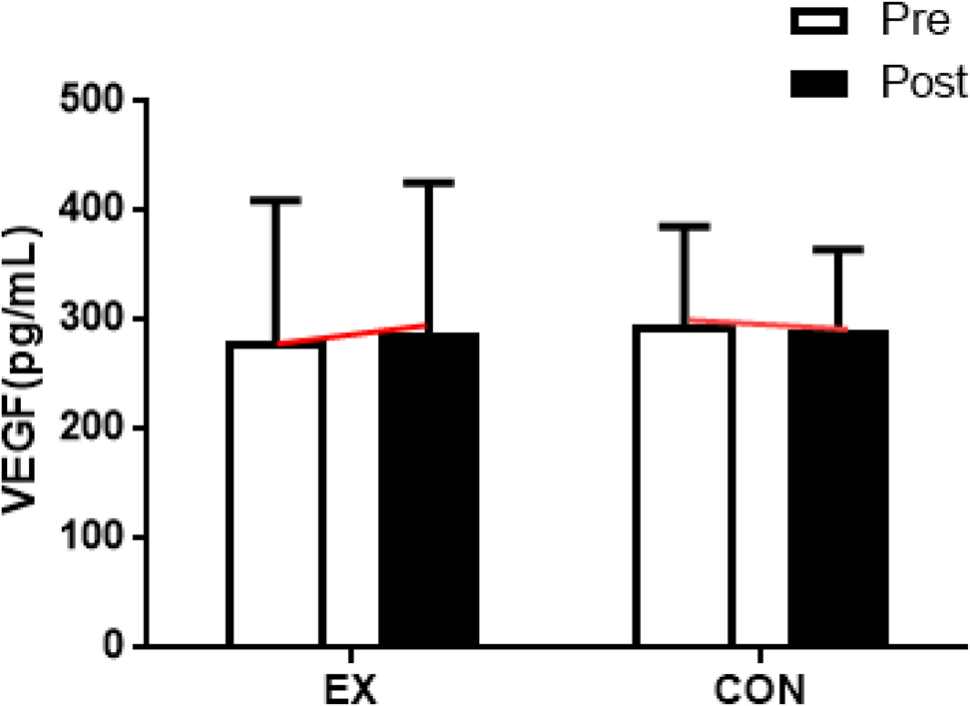
Effect of 12 weeks of moderate intensity walking exercise on VEGF in postmenopausal women with obesity. After the intervention, VEGF levels were not changed compared with the baseline values in all groups. Data are presented as the mean ± standard deviation. *VEGF* Vascular endothelial growth factor.

VEGF showed no significant changes due to walking exercises (interaction effect: *F* = 0.326, *p* = 0.574; time effect: *F* = 0.020, *p* = 0.889; group effect: *F* = 0.037, *p* = 0.849. This is depicted in Fig. 4 and Table S1.

## Discussion

This study was conducted to investigate the effect that twelve weeks of moderate intensity walking exercise has on the body composition, vascular inflammatory factors, and VEGF levels of women to assess whether such exercise can assist with potential postmenopausal obesity. We hypothesized that vascular inflammatory and endothelial growth factor levels would improve after a moderate intensity walking program. Our data confirmed that moderate intensity walking improved body composition and reduced vascular inflammatory factors, but we observed no changes in VEGF levels.

Postmenopausal, rapid physiological and hormonal changes, such as decreased estrogen levels, reduce muscle mass and accelerate fat accumulation^1,28^. As a result, obesity may be induced while the basal metabolic rate decreases^29^. Poehlman^30^ reported that the body composition of women remains constant before menopause, but after menopause, the lean body mass decreases by ~ 3 kg, and body fat increases by ~ 2.5 kg. This increase in body fat is highly correlated with metabolic syndrome and is a risk factor for CVD^31^.

Regular exercise can help improve lean body mass and reduce body fat^32^. A walking program has previously been demonstrated to reduce the body fat percentage in postmenopausal women^33^. This can be ascribed to the use of triacylglycerol in adipose tissue as an energy source during and after exercise, inducing changes in body fat mass^34^ and increasing the energy utilization capacity of fatty tissues^35^. Our study similarly revealed a statistically significant decrease in body fat percentage due to moderate intensity walking exercises. These results suggest that regular moderate intensity walks can help postmenopausal women with obesity prevent and reduce obesity and improve body composition.

When triglycerides and adipose tissue increase in musculature, there is a corresponding increase in inflammatory factors TNF-α and IL-6 in vascular^8,36^. CVD is caused by such vascular inflammation and the formation of blood clots, which can result in arteriosclerosis^37^. TNF-α is a major inflammatory response factor secreted mainly from macrophages and vascular endothelial cells and is primarily involved in the initial stage of inflammation^38^. Arteriosclerosis occurs when excessive TNF-α secretion causes cholesterol accumulation along blood vessel walls^39^. Plasma TNF-α levels can also predict the risk of myocardial infarction^40^, and high serum levels thereof have been reported in obese people^41^. TNF-α serum levels are correlated with those of IL-6^42^. When IL-6 increases, lipoprotein lipase expression is suppressed, inducing hyperlipidemia^43^. Obesity, in particular, is a major factor that increases plasma IL-6 levels, and Vozarava et al*.*^44^ demonstrated that obesity and IL-6 are positively correlated. Conversely, TNF-α^45^ and IL-6^46^ levels decrease after an obese person has lost weight, indicating the effect of regular physical activity^47^.

A previous study showed that TNF-α^48^ and IL-6^49^ decrease with walking exercise. It is known that body fat reduction through exercise induces positive changes in TNF-α and IL-6^50^. In this study, both TNF-α alevels were significantly decreased in participants from the moderate intensity walking exercise group. This confirms that regular moderate intensity walking exercises can reduce body weight and fat^51^ and decrease inflammatory factors in blood vessels and, consequently, may reduce the risk of cardiovascular disease.

Hs-CRP is an inflammatory factor produced in the liver by stimulating inflammatory substances such as TNF-α and IL-6^52^. Therefore, it is a valuable indicator of various inflammatory conditions and the risk of cardiovascular diseases. Obesity increases the serum level hs-CRP^11^, which can result in the formation of thrombi, dysfunction of vascular endothelial cells^53^, and induction of CVD such as atherosclerosis^54,55^. In the Japan Collaborative Cohort study reported that hs-CRP levels were positively associated with CVD^56^. Cohort study of data from 3,119 participants reported that High hs-CRP serum levels strongly associated with the incidence and mortality of cardiovascular disease^57^, whereas decreased hs-CRP levels have been shown to reduce the risk of CVD^58^. Such decreases in hs-CRP levels occur when the body fat and BMI are reduced^59^. Exercise, in particular, has an anti-inflammatory effect that lowers the levels of hs-CRP^60^.

Taghian et al*.*^61^ reported that walking exercises reduce CRP in older women, and this can be due to both a decrease in inflammation and an increase in anti-inflammation through exercise^62^. Furthermore, decreases in hs-CRP have been correlated with decreases in IL-6, which stimulates the secretion of hs-CRP^63,64^. In this study, we observed a significant drop in hs-CRP levels following regular moderate intensity walking exercise. This suggests that moderate intensity walking exercises can help prevent cardiovascular diseases by reducing inflammatory factors.

Obesity disrupts vascular endothelial cell function via heightened inflammation and oxidative stress^65^, and vascular endothelial dysfunction is recognized as a prognostic symptom leading to atherosclerosis^64^. Indeed, increases in adipose tissue have been demonstrated to reduce the density and activity of capillaries in skeletal muscle^66^. VEGF controls the permeability of blood vessels^67^ and plays a vital role in the formation of new capillaries by promoting the proliferation of endothelial cells^68^. Exercise has been shown to increase VEGF expression and, as a result, increase capillary number and density as well as blood flow, thereby promoting angiogenesis and improving the structure and function of blood vessels^69^.

In a previous study, patients with PAD (peripheral arterial disease) experienced an increase in VEGF as a result of treadmill walking^70^. This was ascribed to increases in shear stress and nitric oxide through exercise^71^. On the contrary, Izzicupo et al.^72^ observed no change in VEGF levels in postmenopausal women after participating in 13 weeks of walking exercises. The authors suggested that VEGF fluctuation may be associated with the intensity and nature of exercises^73^. In this study, we similarly encountered no significant difference in VEGF levels between postmenopausal women with obesity who did and did not follow a moderate intensity walking exercise program. However, VEGF showed a tendency to increase slightly in those that exercised. This suggests that continued participation in moderate intensity walking exercises may have a positive effect on VEGF levels.

A potential limitation of this study is that we did not measure direct markers of vascular changes in response to moderate intensity walking. Therefore, further studies need to investigate the direct indices of walking exercise and the relationship between vascular inflammatory factors and VEGF. In addition, there are some limitations in generalizing our findings. First, as the focus of the study was postmenopausal women with obesity, it is difficult to extrapolate the results to men or women of other ages. Second, due to the small sample size, the effect size in this study was confined to a statistical power of 70%, posing a limitation that could potentially influence the outcomes. Therefore, subsequent studies involving a larger number of participants should be conducted to reinforce our findings. Lastly, our study did not implement dietary control to assess the effects of walking exercise while maintaining participants' daily routines. This omission represents a limitation of our research. Therefore, for future studies, incorporating dietary control is important to provide a more specific understanding of the observed changes.

## Conclusion

In summary, moderate intensity walking is an effective therapeutic expected improving percent body fat, TNF- and hs CRP in postmenopausal women with obesity. In addition, since VEGF showed a tendency to increase in participants that exercised, we anticipate that additional research may provide evidence for a similar improvement in VEGF levels as a result of moderate intensity walking exercises. These results support that moderate intensity walking exercise can be expected to prevent CVD by reducing obesity and vascular inflammatory factors in postmenopausal women with obesity.

### Supplementary Information

Supplementary Table S1.

## Data Availability

The datasets used and/or analyzed during the current study are available from the corresponding author on reasonable request.
